# Mitochondrial Genetic Variants Identified to Be Associated with BMI in Adults

**DOI:** 10.1371/journal.pone.0105116

**Published:** 2014-08-25

**Authors:** Antònia Flaquer, Clemens Baumbach, Jennifer Kriebel, Thomas Meitinger, Annette Peters, Melanie Waldenberger, Harald Grallert, Konstantin Strauch

**Affiliations:** 1 Institute of Medical Informatics, Biometry and Epidemiology, Chair of Genetic Epidemiology, Ludwig-Maximilians-Universität Munich, Neuherberg, Germany; 2 Institute of Genetic Epidemiology, Helmholtz Zentrum München - German Research Center for Environmental Health, Neuherberg, Germany; 3 Research Unit of Molecular Epidemiology, Helmholtz Zentrum München - German Research Center for Environmental Health, Neuherberg, Germany; 4 Institute of Human Genetics, Helmholtz Zentrum München-German Research Center for Environmental Health, Neuherberg, Germany; 5 Institute of Epidemiology II, Helmholtz Zentrum München-German Research Center for Environmental Health, Neuherberg, Germany; Kunming Institute of Zoology, Chinese Academy of Sciences, China

## Abstract

It has been suggested that mitochondrial dysfunction plays a role in metabolic disorders including obesity, diabetes, and hypertension. The fact that mitochondrial defects can be accumulated over time as a normal part of aging may explain why some individuals can eat all sorts of foods and remain at normal weight while they are young. However, around the fourth decade of life there is a trend towards “middle-age spread” with weight gain and the body's decreasing ability to metabolize calories efficiently. To test the hypothesis that mitochondrial variants are associated with BMI in adults, we analyzed a total number of 984 mitochondrial single nucleotide polymorphisms (mtSNPs) in a sample of 6,528 individuals participating in the KORA studies. To assess mtSNP association while taking heteroplasmy into account we used the raw signal intensity values measured on the microarray and applied linear regression. Significant results were obtained for 2 mtSNPs located in the Cytochrome c oxidase subunit genes (*MT-CO1*: P_adjusted_ = 0.0140 and *MT-CO3*: P_adjusted_ = 0.0286) and 3 mtSNPs located in the NADH dehydrogenase subunit genes (*MT-ND1*, *MT-ND2* and *MT-ND4L*: P_adjusted_ = 0.0286). Polymorphisms located in the *MT-CO3* and *MT-ND4L* genes have not been associated with BMI or related phenotypes in the past. Our results highlight the importance of the mitochondrial genome among the factors that contribute to the risk of high BMI. Focusing on mitochondrial variants may lead to further insights regarding effects of existing medications, or even to the development of innovative treatments.

## Introduction

Excess of weight is a major risk factor for mortality and morbidity from cardiovascular disease [1], type 2 diabetes [2], and incident cancer [3]. Three million deaths per year worldwide are attributed to overweight and obesity in total [4]. Obesity is a medical condition in which excess body fat has accumulated to the extent that it may have an adverse effect on health [5]. Anyway, the conflation of lean and fat body mass becomes inconsistent in the elderly with both muscle mass and strength begining to decline as part of aging [6]. Changes such as accumulation of intra- and extra-myocellular lipids, improper folding of structural and contractile proteins, and mitochondrial dysfunction are thought to occur with age [7]–[9]. Dysfunctional mitochondria are thought to play a key role in function decline with aging, as the mitochondria are the main producers of both cellular energy and free radicals.

Mitochondria are membrane-bound organelles found in the cytoplasm of almost all eukaryotic cells. Their primary function is to generate large quantities of energy in the form of adenosine triphosphate (ATP). In the early 1960s it was discovered that mitochondria contain their own DNA (mtDNA) of approximately 16.6 kb. It is known to code for 13 subunits of the mitochondrial respiratory chain complexes, 2 ribosomal (rRNA) genes, and 22 transfer RNA (tRNA) genes that are required for mitochondrial protein synthesis. Mitochondria consume oxygen and substrates to generate the vast majority of ATP while producing reactive oxygen species (ROS), also known as free radicals, in the process. An excess of ROS may damage DNA, proteins, and lipids if not rapidly quenched. This damage is termed oxidative stress. It has been recently recognized that oxidative stress is increased (ROS overproduction) in obesity [10], [11] and in fat accumulation in adipocytes [12]. The 13 structural genes are essential for energy production through the process of oxidative phosphorylation (OXPHOS) and generation of ATP. The activity of the OXPHOS system that consists of five enzyme complexes (I–V) can be affected by a number of factors including age and physical training [13]. In addition to supplying ATP and involvement in oxidative stress, mitochondria also participate in other cellular processes, including signal transduction, cell cycle regulation, thermogenesis, and apoptosis. mtDNA is maternally inherited and lacks recombination [14]. One major difference between mtDNA and nuclear DNA (nDNA), which is relevant for the understanding of human diseases, is the *heteroplasmy effect*, which was originally believed to be a rare phenomenon. Because a cell carries many mitochondria, and also the mitochondrial genome has a higher mutation rate than the nuclear genome, there is heterogeneity of the relative frequency of a mtDNA variant within an individual tissue, cell, and even within the same mitochondrion. Therefore, mitochondria are considered heteroplasmic. The clinical expression of some phenotypes is determined by the relative proportion of normal and mutant mitochondrial genomes in different tissues. Mutations of mtDNA are under a growing scientific spotlight and there is increasing evidence that these mutations play a central role in many, if not most, human diseases.

In the past years, genome-wide association studies (GWASs) have identified over 90 common genetic variants associated with BMI in adult samples [15]–[19]. A growing body of research is demonstrating that altered mitochondrial energy production is a major anomaly capable of setting off a chain of metabolic events leading to obesity [20]–[23]. It has also been suggested that particular mtDNA haplogroups might be associated with inefficient energy expenditure [24] and obesity [25]. A mtDNA haplogroup is a collection of mtSNPs at certain genetic loci accumulated throughout human history that could be attributed to genetic drift and/or climate selection [26]. The fact that mitochondrial defects can be accumulated over time as a normal part of aging may explain why some individuals can eat all sorts of foods and remain at normal weight while they are young. However, around the fourth decade of life there is a trend towards “middle-age spread” with weight gain and the body's decreasing ability to metabolize calories efficiently. In two newly published studies in animals, researchers show how the dynamics of the mitochondria play a key role in the body's ability to control weight gain [27], [28]. Hence, whether one becomes obese or remains lean can depend on the dynamics of the mitochondria, the body's energy-producing “battery”.

The purpose of the current study was to conduct a mitochondrial GWAS to identify genetic variants influencing BMI. In particular, we tested 984 mtSNPs in a sample of 6,528 adults, aged 24–85 years.

## Results

**Figure 1:**
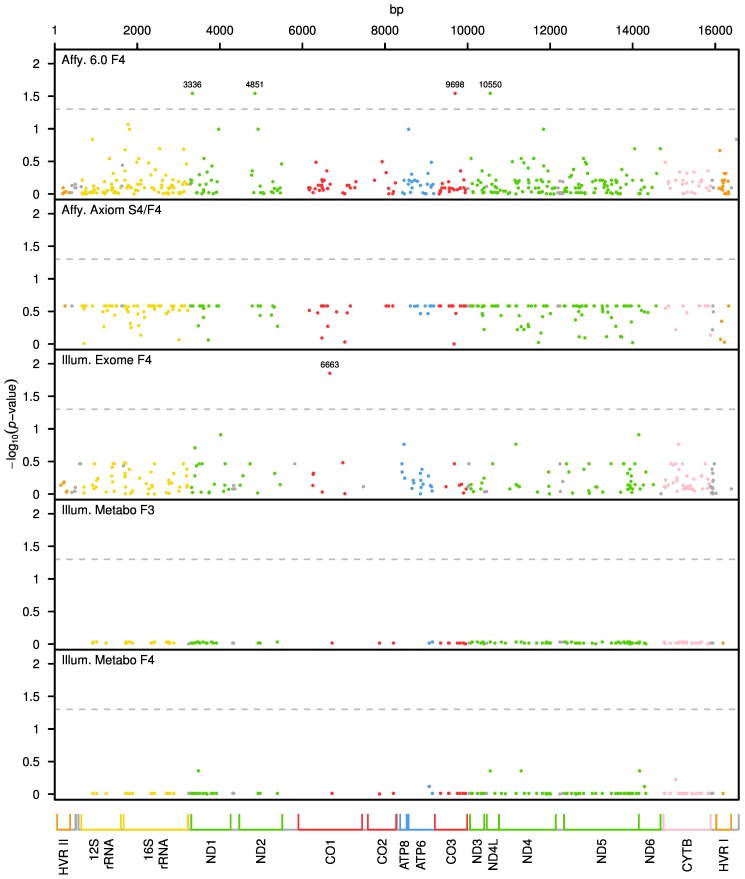
Mitochondrial genome-wide *P* values after adjusting for multiple testing. On the y axis, adjusted p-values transformed into the negative of the base 10 logarithm, −log_10_(p-value), are shown. The x-axis represents the mitochondrial genome, displaying the position and relative size of each of the 13 major mitochondrial genes, 12S and 16S rRNAs, hypervariable region 1 (HVRI), hypervariable region 2 (HVR II) as well as the position of the 22 tRNAs (gray). The dashed lines show the critical values of the pointwise significance level corresponding to an FDR of 0.05.

We performed mitochondrial genome wide association analysis separately for each genotyping chip and corresponding sample (see Table 1). After QC, a total number of 984 mtSNPs were included in the analysis. To assess mtSNP association while taking heteroplasmy into account, we used the raw signal intensity values that were measured on the microarray and applied the linear regression method as described in the Materials and Methods section. The resulting p-values after adjustment for multiple testing are plotted in Figure 1 for each genotyping chip. The x-axis represents the mitochondrial genome, showing the position and relative size of each of the 13 major mitochondrial genes.

**Table 1:** Summary of the quality control.

Chip	mtSNPs	mtSNPs excluded UB no_B38		Study: Sample size	I_SNP_	I_tot_	Intensity Ratio Outliers
Affy. 6.0	**411**	0	54	F4:1,646	3	4,059,036	217 (<0.05%)
Affy. Axiom	**221**	31	0	S4/F4:3,530	4	6,241,040	62 (<0.05%)
Illum. Exome	**226**	0	0	F4: 2,721	1	1,229,892	123 (<0.05%)
Illum. Metabo	**126**	0	9	F3:2,889	1	728,028	96 (<0.05%)
				F4:2,817		709,884	99 (<0.05%)

The number of **mtSNPs** refers to the SNPs that passed QC and were included in the analysis. Several mtSNPs were excluded due to the upper bound cut-off (**UB**) or because the basepair position was not available in Build 38 (**no_B38**). Sample size is based on the particular chip. One person may be present on more than one chip. *I_SNP_* stands for the number of intensity measures per allele. *I_tot_* represents the total number of intensity measures in the sample (I_SNP_*2*sample-size*mtSNPs).

Four and one mtSNPs were significant for Affymetrix 6.0 and Illumina Exome chip, respectively. No significant mtSNPs were observed when analysing the Axiom and Metabochip. The association results that remained significant after adjustment for multiple testing (P_adjusted_≤0.05) are presented in Table 2. The two most significant mtSNPs associated with BMI are located in the *Cytochrome c oxidase* 1 and 3 (*MT-CO1*_mt6663A→G_ and *MT-CO3*_mt9698T→C_) genes of complex IV, with associated adjusted p-values of 0.014 and 0.029, respectively. Another three locations of *NADH subunit dehydrogenase* genes of complex I were also significant (*MT-ND1*_mt3336T→G_: P_adjusted_ = 0.029; *MT-ND2*_mt4851C→T_: P_adjusted_ = 0.029; *MT-ND4L*_mt10550A→G_: P_adjusted_ = 0.029). In all significant mtSNPs the estimates of β_sex_ and β_age_ were significant and positive, indicating a significantly higher BMI in males and with older age. All five significant mtSNPs were present only on one chip without overlapping, i.e., they were not shared by the other chips.

**Table 2:** Summary of significant mtSNPs.

Chip	Bp	Alleles rs_number	Type of mutation	β_SNP_	P_nominal_	P_adj_	Protein: *Gene*
Affy6.0	3336	T→G rs28416101	missense	−0.23	2.6×10^−04^	0.03	ND1: *MT-ND1*; subunit of NADH dehydrogenase (complex I)
Affy6.0	4851	C→T rs28413696	synonymous	−1.11	1.7×10^−04^	0.03	ND2: *MT-ND2*; ubunit of NADH dehydrogenase (complex I)
Affy6.0	9698	T→C rs9743	synonymous	−0.23	2.2×10^−04^	0.03	COIII: *MT-CO3*; subunit of cytochrome c oxidase (complex IV)
Affy6.0	10550	A→G rs28358280	synonymous	−0.31	2.8×10^−04^	0.03	ND4L: *MT-ND4L*; subunit of NADH dehydrogenase (complex I)
Illum. Exome	6663	A→G rs200784106	missense	−0.55	6.2×10^−05^	0.014	COI: *MT-CO1*; subunit of cytochrome c oxidase (complex IV)

Genomic position in base pairs (bp), alleles, rs_number, and type of mutation are based on the NCBI dbSNP GRCh38 human genome assembly (rCRS, GeneBank ID J01415.2). Alleles are given in terms of major→minor allele. An estimated effect size (β_SNP_)<0 indicates that the minor allele increases BMI. Nominal p-values and adjusted p-values are provided.

A negative parameter estimate for the mtSNP (β_SNP_<0) indicates that the risk allele is the minor allele while a β_SNP_>0 indicates that the risk allele is the major allele. Taking the most strongly associated variant mt6663A→G in the *MT-CO1* gene, based on our estimate (β_mt6663A→G_ = −0.55±0.1) BMI increases when the ratio of alleles A and G decreases, i.e., an increase in heteroplamy with the G allele at this locus leads to an increased BMI. The same applies to the other mtSNPs; e.g. having mt10550A→G heteroplasmy with more G than A alleles results in a higher BMI than having only A alleles. The estimates of the model parameters for each significant mtSNP are provided in Table S1.

## Discussion

Excess of weight is a medical condition in which excess body fat has accumulated to the extent that it may have an adverse effect on health, leading to reduced life expectancy and/or increased health problems. The possible role of mitochondria in the development of obesity is mainly concerned with the efficacy of mitochondrial energy provision [29], [30]. Apparently, the balance between energy conservation into the ATP molecule and energy dissipation in the form of heat loss thus represents the key metabolic control, which is crucial for possible development of obesity. Obesity, as well as fat accumulation in adipocytes, has been related to an increased ROS production in human mitochondria [10]–[12]. In the current study, in which we examined association between BMI and mtSNPs, we identified two mtSNPs (mt6663A→G, mt9698T→C) located in Cytochrome c oxidase subunits (*MT-CO1* and *MT-CO3*) and three mtSNPs (mt3336T→G, mt4851C→T, mt10550A→G) located in NADH subunits (*MT-ND1*, *MT-ND2*, and *MT-ND4L*), that were significantly associated with BMI after adjusting for multiple testing. Although mtDNA is transmitted from mother to offspring unchanged (lack of recombination), due to the higher mutation rate in mtDNA compared to nDNA, many mutations have occurred only recently rather than many generations ago, and even in the current generation. For that reason pairwise LD is reduced, and when one mtSNP is significant it is not expected that the neighboring mtSNPs are also significant.

### *MT-CO1*_mt6663A→G_ and *MT-CO3*_mt9698T→C_

***Mitochondrially encoded cytochrome c oxidase subunits, complex IV***, is a key oxidative enzyme regarded as one of the major regulation sites for the OXPHOS system, controlled by both nDNA and mtDNA. Its catalytic activity is primarily determined by 3 of the 13 subunits which are encoded by the mtDNA (*MT-CO1*, *MT-CO2*, and *MT-CO3*) [31]. The loss of function of this enzyme has been suggested to trigger ROS production, although the increase in radical accumulation rests not with the ETC but with non-mitochondrial sources [32]. However, the function of each subunit and the molecular mechanism behind the regulation on the activity of this important protein complex are largely unknown [33]. The interesting finding that suppression of cytochrome c oxidase activity also sensitizes cells to death signals [33] could shed some light on the role of defective cytochrome c oxidase in aging and age-related diseases as an increase of BMI with age.

### *MT-ND1*_mt3336T→G_, *MT-ND2*_mt4851C→T_, and *MT-ND4L*_mt10550A→G_

***Mitochondrially encoded NADH dehydrogenase subunit genes, complex I***, is the first enzyme in the mitochondrial respiratory chain. It extracts energy from NADH, produced by the oxidation of sugars and fats, and traps the energy in a potential difference or voltage across the mitochondrial inner membrane. The potential difference is used to power the synthesis of ATP. Because NADH dehydrogenase is central to energy production in the cell, its malfunction may result in a wide range of metabolic disorders. Some of them are due to mutations in the mitochondrial genome, while others, which result from a decrease in the activity of complex I, or an increase in the production of ROS, are not yet well understood.

*MT-ND1* and *MT-ND2* genes were previously reported to be related with body fat mass and intramuscular fat in animals [23], [34], [35]. The significant findings in our study at mt3336T→G (*MT-ND1*) and mt4851C→T (*MT-ND2*) have associated effect sizes estimated to be −0.23 and −1.11, respectively. In other words, increasing the number of T alleles (or decreasing G alleles) at mt3336 in an individual will help to reduce the BMI, while increasing the number of T alleles (or decreasing C alleles) at mt4851 will contribute to increase BMI. In this study we identified for the first time a significant association between *MT-ND4L* (mt10550A→G) and BMI, with an estimated effect size of −0.31. Therefore, the G allele is the one to be related with high BMI values, i.e., people with heteroplasmy having a large number of G alleles will have a higher BMI than people with a smaller number of G alleles. *MT-ND4L* is one of the 7 mtDNA-encoded subunits included among the approximately 41 polypeptides of respiratory complex I. *MT-ND4L* is probably a component of the hydrophobic protein fragment of the complex [36].

While associations of the *MT-CO1* gene with intramuscular fat in animals have been reported [35], no significant findings have been reported so far for the *MT-CO3* gene with BMI, nor with other BMI-related phenotypes. Liu et al. [37] suggested an effect of mtSNPs, including *MT-CO2*, *MT-ND1*, and *MT-ND2* genes, on BMI but this effect failed to reach significance after adjusting for multiple testing. Similarly, Saxena et al. [38] detected nominal signals of association between BMI and mtSNPs, including *MT-ND1*, *MT-ND2*, and *MT-ND3* genes; however after adjusting for multiple testing these associations disappeared.

Recently, Knoll et al., 2014 [39] did not find association between mtSNPs and obesity in children. However, this may be because heteroplasmy was not included in their study or different mtSNPs might be relevant for children than adults with regard to BMI.

Two of the significant variants *MT-ND1*_mt3336T→G_ and *MT-CO1*_mt6663A→G_ are missense mutations which lead to an amino acid change, thus being a non-synonymous variant. So, individuals with an excess of missense mutations may carry an appreciable fraction of an altered protein that is responsible for an elevated BMI. The other three significant variants *MT-ND2*_mt4851C→T_, *MT-CO3*_mt9698T→C_, and *MT-ND4L*_mt10550A→G_ are synonymous mutations, i.e., they code for the same amino acid. How an excess of synonymous mutations at these loci could impact BMI needs further investigation, since the single nucleotide change leads to an unchanged protein. However, different codons might lead to differential protein expression levels. Two of the synonymous variants, mt9698C and mt10550G, are present in the European mtDNA haplogroups U8 and K, respectively. However, to date only haplogroup T has been suggested as a risk factor for obesity [24].

These results together with previously published findings suggest that genetic variation in NADH dehydrogenase and cytochrome c is a risk factor for several metabolic disorders, including BMI and BMI-related phenotypes. Multiple lines of evidence suggest that mitochondrial dysfunction as well as altered mitochondrial genome expression, particularly of genes encoding complex I–IV may play a role in BMI. Searching for previous suggested candidate mtSNPs associated with BMI or related phenotypes has yielded a small number of positive findings, possibly due to low sample sizes or the lack of taking heteroplasmy into account in the analysis. In this study we have considered the heteroplasmy effect and found mtSNPs in candidate gene regions to be significantly associated with BMI that previously were suggested to be implicated in obesity, body fat mass, or intramuscular fat (*MT-ND1*, *MT-ND2*, and *MT-CO1*). In addition, two novel susceptibility mtSNPs in the *MT-ND4L* and in the *MT-CO3* regions have been identified. In both polymorphisms heteroplasmic individuals towards a larger number of the respective minor allele increases the risk of developing a high BMI.

The regression models for all significant mtSNPs identified in this study also corroborate the generally acknowledged fact that males have slightly but significant higher BMI values than females and BMI also increases significantly with older age. With repond to sex, this effect is to be expected due to the fact that adult males and females with the same BMI are classified identically in regard to whether their weight is normal. However, even though men may have slightly higher BMI values than women, this does not necessarily mean that they are more overweight. For instance, women typically have more body fat than men, whereas men typically have more muscle than women, and muscle has a much greater density (it takes up less volume than an equal mass of fat). It is also known that BMI is largely influenced by age in adults [40], [41]. It has been suggested that BMI increases linearly with age in women, but in men it increases with age in two stages – a more intensive rise between age 20–40 and a much slower increase between age 40–60 [42]. High BMI can occur at any age, but with both muscle mass and strength beginning to decline around the age of 40, an increased BMI at this age may be related to mitochondrial dysfunction.

## Conclusion

In summary, our study reports genetic variants located in the cytochrome c subunits 1, 3, and in the NADH dehydrogenase subunits 1, 2, and 4L of the mtDNA that are significantly associated with BMI in adults. Heteroplasmy in these variants towards the minor allele increases the risk of developing high BMI. Based on these findings we hypothesize that common metabolic diseases as obesity or high BMI are attributable at least in part to mitochondrial polymorphisms. Animal and human data consistently show that mitochondria are altered in aging, leading to increased mutations in mtDNA, decreased expression of some mitochondrial proteins, reduced enzyme activity, and altered respiration with reduced maximal capacity in sedentary adults. Since the primary role of mitochondria is to produce ATP to maintain the energy status of the cell, shifts in respiratory activity and capacity can lower the membrane potential, reduce cellular ATP concentration, and signal cellular apoptotic events. Increased apoptosis without correspondingly increased protein synthesis will eventually lead to net muscle fiber loss. All of these factors are likely to contribute to age-associated BMI, and mounting evidence suggests that most of these age-related changes can be either prevented or attenuated through increased physical activity. There are some thoughts that the accumulation of oxidative damage caused by long-term ROS production is responsible for these changes with age. Cytochrome c and NADH dehydrogenase complexes are both involved in the regulation of mitochondrial ROS. Indeed, blocking ROS formation in animals by the targeted expression of antioxidant enzymes ameliorates age-associated dysfunction and returns mitochondrial parameters to those of young animals [43]. Whether this is also true for humans remains to be shown. However, behavioral factors that may improve mitochondrial function and BMI control, such as physical activity and caloric restriction, also reduce ROS production and increase antioxidant defenses. Although physical activity training in elderly adults probably does not completely reverse the primary effects of aging, it may clearly reduce the rate of mitochondrial decline and attenuate the aging processes [44].

These findings highlight the important role of the mtDNA among the factors that contribute to the risk of high BMI in adults and suggest that variants in the mitochondrial genome may be more important than has previously been suspected. Therefore, a challenge for possible novel therapeutic strategies might be the further elucidation of intracellular ROS signaling pathways for effective therapeutic gain.

## Materials and Methods

### Study design and population

The Cooperative Health Research in the Region of Augsburg (KORA) study is a series of independent population-based epidemiological surveys and follow-up studies of participants living in the region of Augsburg, in southern Germany, in a mixed urban and rural area with demographic and socioeconomic characteristics roughly reflecting those of an average central European population. All participants are residents of German nationality identified through the registration office and written informed consent was obtained from each participant [45]. The study was approved by the local ethics committee (Bayerische Landesärztekammer). All participants filled in a self-administrated questionnaire and underwent a standardized personal interview and an extensive medical examination. The study design, sampling method, and data collection have been described in detail elsewhere [46]. The most recent KORA studies are KORA S4 (1999–2001) with its follow-up KORA F4 (2006–2008) and KORA S3 (1994–1995) with its follow-up KORA F3 (2004–2005). The present study includes data of the studies F3, F4, and additionally from those individuals of S4 that did not participate in F4, including a total number of 6,528 unrelated individuals. In order to avoid confounding with insulin-dependent diabetes mellitus, 247 individuals diagnosed as diabetic were not included in the study.

### BMI phenotype

The body mass index (BMI), or Quetelet index (devised between 1830 and 1850 by the Belgian polymath Adolphe Quetelet), is a measure for human body shape defined as the individual's body weight divided by the square of the height (kg/m^2^). All participants were subjected to several medical examinations including BMI measurement.

### Genotyping

**Figure 2:**
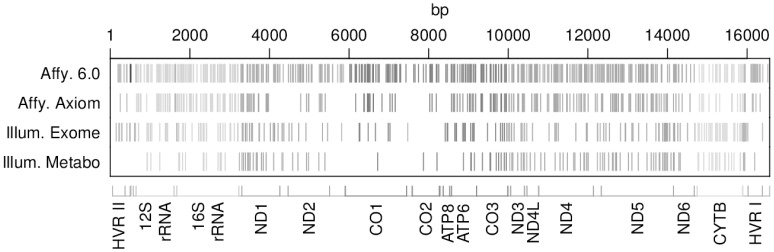
Coverage of the mitochondrial genome provided by each genotyping chip. Each vertical bar stands for one mtSNP. The x-axis represents the mitochondrial genome, displaying the position and relative size of each of the 13 major mitochondrial genes, 12S and 16S rRNAs, hypervariable region 1 (HVRI), hypervariable region 2 (HVR II) as well as the position of the 22 tRNAs.

DNA was extracted from full blood after the blood draw and then stored at −80°C. Genotyping was performed using different platforms such as the Affymetrix 6.0 GeneChip array (KORA F4), Illumina MetaboChip 200K (KORA F3 and F4), Illumina Human Exome Beadchip array (KORA F4), and Affymetrix Axiom chip array (KORA S4 and F4). A total number of 6,528 individuals were genotyped. Some individuals of KORA F4 were present on more than one chip, some of them (1,549) were included on all chips. Only single-nucleotide polymorphisms located in the mitochondrial genome (mtSNPs) were considered in this study. Genotyping data and samples are summarized in Table 3. Most of the covered mtSNPs have distinct positions identified by different chips. Although the Affymetrix 6.0 is the one containing the largest number of mtSNPs some regions are not well covered. The Illumina Metabochip contains the smallest number of mtSNPs and many regions are uncovered, especially the hypervariable regions (HVRI and HVRII) as well as the CO1 and CO2 genes. However, when all chips are considered together, good overall coverage of the mitochondrial genome is obtained (Figure 2).

**Table 3:** Distribution of characteristics of the study population.

Chip	mtSNP	KORA	Individuals (Males/Females)	Mean age Males/Females	Mean BMI Males/Females
Affy. 6.0	465	F4	1646 (788/858)	60.4±8.8/60.1±8.7	28.2±4.3/27.5±4.9
Affy. Axiom	252	S4/F4	3530 (1702/1828)	54.1±14.2/53.6±13.9	27.7±4.2/26.9±5.1
Illum. Exome	226	F4	2721 (1294/1427)	55.5±13.2/54.8±13.1	27.7±4.0/26.9±5.1
Illum. Metabochip	135	F3	2889 (1393/1496)	56.6±12.9/56.1±12.5	27.8±3.7/27.0±5.0
		F4	2817 (1338/1479)	55.5±13.2/54.9±13.1	27.7±4.1/27.0±5.1

The mtSNPs column shows the number of mitochondrial SNPs provided by each chip. Distributions are presented as means ± standard deviation.

### Genotype calling

Current SNP microarrays are able to genotype more than a million SNPs simultaneously. There are some statistical issues such as the normalization of raw intensities, background correction, outlier detection, and allele calling (genotyping) that are shared between different microarray platforms irrespective of chip technology and design principles. The most commonly used platforms, Affymetrix and Illumina, represent a SNP by a number of probes (Affymetrix) or beads (Illumina) for each of the two alleles. The probes/beads have different affinities, depending on the DNA sequence they target. A first step in many algorithms for genotyping is to cluster the probe intensities for each allele and SNP, and in a second step to make a call based on the clustered intensities.

The allele frequency, one of the most important population indices used in genetics, denotes the relative frequency of a certain allele at a marker locus. There are two kinds of allele frequencies: *individual-level* that represents the within-individual relative frequency of alleles and its standard error reflects inter-cell variability in an individual, and *population-level* that refers to the within-population relative frequency of alleles and its standard error reflects inter-individual variability in a population [47]. Using SNPs, both individual and population allele frequencies can be estimated using an allele counting approach (genotype-based) and using an intensity measuring approach (intensity based). A problem arises, however, when dealing with mtSNPs due to the heteroplasmy effect, causing heterogeneous mtDNA in the sense that different mitochondria can have different genotypes, such that a genotype at an mtDNA locus may not be restricted to 0, 1, or 2 minor alleles. This issue affects the possibility of estimating genotypes and makes calling algorithms useless. Therefore, whenever one intends to identify susceptibility genes located in the mtDNA we recommend accounting for heteroplasmy using individual-level allele frequencies obtained from the intensity values.

### Quality control

First a visual check was performed plotting the intensity measures of each mtSNP separately for each chip. In this step only the Axiom chip displayed anomalies in some intensity values. There seems to be an upper bound for the intensity values at an intensity of 4095. As a result some mtSNPs had a significant fraction of either their A or B intensities squeezed into the region just below that upper bound; apparently without the bound those intensities would have taken higher values. Since intensities close to the cut-off value of 4095 cannot be trusted, we proceeded as described in the Text S1.

After the visual check, normalization of microarray data and outlier corrections were performed separately for each chip as described in Text S1. A summary of the quality control results is given in Table 1.

### Statistical methods

To approach the heteroplasmy present in the mitochondria we used the raw signals of luminous intensity, where every measurement is associated with a specific mtSNP and represents one of its alleles [48], [49]. The number of measures *n* per mtSNP depends on the vendor-specific technology employed on the genotyping chip. At the very least there have to be two signals, one for each of the two alleles. Often, however, the chip design includes more than one measurement per SNP and allele. That is, for every individual and SNP we have intensity measurements (A_1_, B_1_), …, (A_n_, B_n_) with n> = 1 where A_i_ and B_i_ represent the intensities of the two alleles A and B. The best way to assess the association of BMI with the mtSNP intensities at a given age is to apply linear regression analysis using BMI as quantitative response variable. The mtSNP enters the model as a covariate via the log_2_-transformed intensity ratio, log_2_(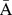/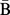), where 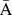 and 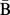 denote the mean intensity, or single measure in case of n = 1, for the A allele and B allele, respectively. To improve the convergence properties of the model estimates we center this variable (z = log_2_(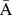/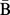)−μ) as well as the additional quantitative covariate age at examination, i.e., the age at the time that the mtSNPs and BMI were measured. Sex is also introduced in the model as a covariate. The linear regression model for the *ith*-mtSNP is:
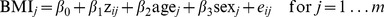
where *j* denotes the individual and *m* is the sample size. The aim of this study is to evaluate how BMI is influenced by mitochondrial variants taking into account the age for a particular person on the phenotype BMI, and therefore the model adjusts for individual age differences in the study population. Using this model it is not possible to make any claims based on the effect of age on heteroplasmy at the particular mtSNP. However, due to the adjustment by age on BMI in the model, we can be sure that in case of positive findings the BMI change is in fact caused by variations of heteroplasmy at the particular mtSNP, and not by differences in age that jointly influence BMI and heteroplasmy, without an effect of heteroplasmy on BMI.

P-values were obtained from the Wald test, which is based on the asymptotic normality of the regression coefficient estimates, and corrected for multiple comparisons applying the Benjamini-Hochberg false discovery rate (FDR) using the R function p.adjust [50]. The resulting adjusted P-values are also known as “Q-values”. Each type of genotyping chip needs to be analyzed separately because different chips make use of different technologies, even between chips of the same manufacturer. The two Affymetrix chips, Affy6.0 and Axiom, share 170 positions and Illumina chips, Exome and Metabochip, share 44 positions. Only 9 positions are common to all four chips. Overlapping mtSNPs were analysed separately for each respective genotyping chip, giving in this way the opportunity of validation in the case of significant results. All the analyses were performed with the R statistical software [51].

## Supporting Information

Figure S1 **Examples of intensities affected by the cut-off.**
(TIFF)

Figure S2 **Resulting p-values from the 1^st^ analysis (left side) and 2^nd^ analysis (right side).**
(TIFF)

Table S1 **Estimates of the model parameters for mtSNPs with a P_nominal_< = 0.01.** Genomic position in base pairs (bp), alleles, rs_number, and point mutation are based on the NCBI dbSNP GRCh38 human genome assembly (rCRS, GeneBank ID J01415.2). Alleles are given in terms of major→minor allele. An estimated effect size (β_SNP_)<0 indicates that the risk allele is the minor allele. Nominal p-values and adjusted p-values are provided. μ: mean of log_2_(A/B). Covariate sex baseline: male.
(DOC)

Text S1 **Quality control.**
(DOCX)

## References

[1] Wormser D, Kaptoge S, Di Angelantonio E, Wood AM, Pennells L, et al (2011) Separate and combined associations of body-mass index and abdominal adiposity with cardiovascular disease: collaborative analysis of 58 prospective studies. Lancet 377: 1085–1095. 21397319 10.1016/S0140-6736(11)60105-0 PMC3145074

[2] Vazquez G, Duval S, Jacobs DR Jr, Silventoinen K (2007) Comparison of body mass index, waist circumference, and waist/hip ratio in predicting incident diabetes: a meta-analysis. Epidemiol Rev 29: 115–128. 17494056 10.1093/epirev/mxm008

[3] Renehan AG, Tyson M, Egger M, Heller RF, Zwahlen M (2008) Body-mass index and incidence of cancer: a systematic review and meta-analysis of prospective observational studies. Lancet 371: 569–578. 18280327 10.1016/S0140-6736(08)60269-X

[4] World Health Organization (2010) International Statistical Classification of Diseases and Health Related Problems. Geneva: World Health Organization.

[5] Haslam DW, James WP (2005) Obesity. Lancet 366: 1197–1209. 16198769 10.1016/S0140-6736(05)67483-1

[6] Frontera WR, Hughes VA, Lutz KJ, Evans WJ (1991) A cross-sectional study of muscle strength and mass in 45- to 78-yr-old men and women. J Appl Physiol (1985) 71: 644–650. 1938738 10.1152/jappl.1991.71.2.644

[7] Cree MG, Newcomer BR, Katsanos CS, Sheffield-Moore M, Chinkes D, et al (2004) Intramuscular and liver triglycerides are increased in the elderly. J Clin Endocrinol Metab 89: 3864–3871. 15292319 10.1210/jc.2003-031986

[8] Hipkiss AR (2010) Mitochondrial Dysfunction, Proteotoxicity, and Aging: Causes or Effects, and the Possible Impact of NAD+-Controlled Protein Glycation. In: Makowski GS, editor. Advances in Clinical Chemistry. Burlington: Academic Press. pp. 123–150. 20521444

[9] Johannsen DL, Conley KE, Bajpeyi S, Punyanitya M, Gallagher D, et al (2012) Ectopic lipid accumulation and reduced glucose tolerance in elderly adults are accompanied by altered skeletal muscle mitochondrial activity. J Clin Endocrinol Metab 97: 242–250. 22049170 10.1210/jc.2011-1798 PMC3251940

[10] Furukawa S, Fujita T, Shimabukuro M, Iwaki M, Yamada Y, et al (2004) Increased oxidative stress in obesity and its impact on metabolic syndrome. J Clin Invest 114: 1752–1761. 15599400 10.1172/JCI21625 PMC535065

[11] Sankhla M, Sharma TK, Mathur K, Rathor JS, Butolia V, et al (2012) Relationship of oxidative stress with obesity and its role in obesity induced metabolic syndrome. Clin Lab 58: 385–392. 22783566

[12] Lee H, Lee YJ, Choi H, Ko EH, Kim JW (2009) Reactive oxygen species facilitate adipocyte differentiation by accelerating mitotic clonal expansion. J Biol Chem 284: 10601–10609. 19237544 10.1074/jbc.M808742200 PMC2667747

[13] Chretien D, Rustin P, Bourgeron T, Rotig A, Saudubray JM, et al (1994) Reference charts for respiratory chain activities in human tissues. Clin Chim Acta 228: 53–70. 7955429 10.1016/0009-8981(94)90056-6

[14] Elson JL, Andrews RM, Chinnery PF, Lightowlers RN, Turnbull DM, et al (2001) Analysis of European mtDNAs for recombination. Am J Hum Genet 68: 145–153. 11115380 10.1086/316938 PMC1234908

[15] Berndt SI, Gustafsson S, Maegi R, Ganna A, Wheeler E, et al (2013) Genome-wide meta-analysis identifies 11 new loci for anthropometric traits and provides insights into genetic architecture. Nature genetics 45: 501–512. 23563607 10.1038/ng.2606 PMC3973018

[16] Okada Y, Kubo M, Ohmiya H, Takahashi A, Kumasaka N, et al (2012) Common variants at CDKAL1 and KLF9 are associated with body mass index in east Asian populations. Nat Genet 44: 302–306. 22344221 10.1038/ng.1086 PMC3838874

[17] Speliotes EK, Willer CJ, Berndt SI, Monda KL, Thorleifsson G, et al (2010) Association analyses of 249,796 individuals reveal 18 new loci associated with body mass index. Nat Genet 42: 937–948. 20935630 10.1038/ng.686 PMC3014648

[18] Thorleifsson G, Walters GB, Gudbjartsson DF, Steinthorsdottir V, Sulem P, et al (2009) Genome-wide association yields new sequence variants at seven loci that associate with measures of obesity. Nat Genet 41: 18–24. 19079260 10.1038/ng.274

[19] Wen W, Cho YS, Zheng W, Dorajoo R, Kato N, et al (2012) Meta-analysis identifies common variants associated with body mass index in east Asians. Nat Genet 44: 307–311. 22344219 10.1038/ng.1087 PMC3288728

[20] Bournat JC, Brown CW (2010) Mitochondrial dysfunction in obesity. Curr Opin Endocrinol Diabetes Obes 17: 446–452. 20585248 10.1097/MED.0b013e32833c3026 PMC5001554

[21] Ritov VB, Menshikova EV, Azuma K, Wood R, Toledo FG, et al (2010) Deficiency of electron transport chain in human skeletal muscle mitochondria in type 2 diabetes mellitus and obesity. Am J Physiol Endocrinol Metab 298: E49–58. 19887598 10.1152/ajpendo.00317.2009 PMC2806111

[22] Wortmann SB, Zweers-van Essen H, Rodenburg RJ, van den Heuvel LP, de Vries MC, et al (2009) Mitochondrial energy production correlates with the age-related BMI. Pediatr Res 65: 103–108. 19096353 10.1203/PDR.0b013e31818d1c8a

[23] Yang TL, Guo Y, Shen H, Lei SF, Liu YJ, et al (2011) Genetic association study of common mitochondrial variants on body fat mass. PLoS One 6: e21595. 21747914 10.1371/journal.pone.0021595 PMC3126834

[24] Nardelli C, Labruna G, Liguori R, Mazzaccara C, Ferrigno M, et al (2013) Haplogroup T is an obesity risk factor: mitochondrial DNA haplotyping in a morbid obese population from southern Italy. Biomed Res Int 2013: 631082. 23936828 10.1155/2013/631082 PMC3713591

[25] Pichaud N, Ballard JW, Tanguay RM, Blier PU (2012) Naturally occurring mitochondrial DNA haplotypes exhibit metabolic differences: insight into functional properties of mitochondria. Evolution 66: 3189–3197. 23025608 10.1111/j.1558-5646.2012.01683.x

[26] Mishmar D, Ruiz-Pesini E, Golik P, Macaulay V, Clark AG, et al (2003) Natural selection shaped regional mtDNA variation in humans. Proc Natl Acad Sci U S A 100: 171–176. 12509511 10.1073/pnas.0136972100 PMC140917

[27] Dietrich MO, Liu ZW, Horvath TL (2013) Mitochondrial dynamics controlled by mitofusins regulate Agrp neuronal activity and diet-induced obesity. Cell 155: 188–199. 24074868 10.1016/j.cell.2013.09.004 PMC4142434

[28] Schneeberger M, Dietrich MO, Sebastian D, Imbernon M, Castano C, et al (2013) Mitofusin 2 in POMC neurons connects ER stress with leptin resistance and energy imbalance. Cell 155: 172–187. 24074867 10.1016/j.cell.2013.09.003 PMC3839088

[29] Brady LJ, Brady PS, Romsos DR, Hoppel CL (1985) Elevated hepatic mitochondrial and peroxisomal oxidative capacities in fed and starved adult obese (ob/ob) mice. Biochem J 231: 439–444. 4062906 10.1042/bj2310439 PMC1152765

[30] Katyare SS, Howland JL (1978) Enhanced oxidative metabolism in liver mitochondria from genetically obese mice. Arch Biochem Biophys 188: 15–20. 677888 10.1016/0003-9861(78)90349-1

[31] Capaldi RA (1990) Structure and function of cytochrome c oxidase. Annu Rev Biochem 59: 569–596. 2165384 10.1146/annurev.bi.59.070190.003033

[32] Leadsham JE, Sanders G, Giannaki S, Bastow EL, Hutton R, et al (2013) Loss of cytochrome c oxidase promotes RAS-dependent ROS production from the ER resident NADPH oxidase, Yno1p, in yeast. Cell Metab 18: 279–286. 23931758 10.1016/j.cmet.2013.07.005

[33] Li Y, Park JS, Deng JH, Bai Y (2006) Cytochrome c oxidase subunit IV is essential for assembly and respiratory function of the enzyme complex. J Bioenerg Biomembr 38: 283–291. 17091399 10.1007/s10863-006-9052-z PMC1885940

[34] Guo LJ, Oshida Y, Fuku N, Takeyasu T, Fujita Y, et al (2005) Mitochondrial genome polymorphisms associated with type-2 diabetes or obesity. Mitochondrion 5: 15–33. 16060290 10.1016/j.mito.2004.09.001

[35] Lee SH, Kim SC, Choi BH, Lim D, Kim NK, et al (2012) mt-COX1, mt-ND1 and CREBP are indicators of intramuscular fat content in Hanwoo (Korean cattle). Livestock Science 146: 160–167.

[36] Ragan CI (1987) Structure of NADH-ubiquinone reductase (complex I). Curr Top Bioenergy 15: 1–36.

[37] Liu C, Yang Q, Hwang SJ, Sun F, Johnson AD, et al (2012) Association of genetic variation in the mitochondrial genome with blood pressure and metabolic traits. Hypertension 60: 949–956. 22949535 10.1161/HYPERTENSIONAHA.112.196519 PMC3753106

[38] Saxena R, de Bakker PI, Singer K, Mootha V, Burtt N, et al (2006) Comprehensive association testing of common mitochondrial DNA variation in metabolic disease. Am J Hum Genet 79: 54–61. 16773565 10.1086/504926 PMC1474138

[39] Knoll N, Jarick I, Volckmar AL, Klingenspor M, Illig T, et al (2014) Mitochondrial DNA variants in obesity. PLoS One 9: e94882. 24788344 10.1371/journal.pone.0094882 PMC4008486

[40] Nube M, Asenso-Okyere WK, van den Boom GJ (1998) Body mass index as indicator of standard of living in developing countries. Eur J Clin Nutr 52: 136–144. 9505160 10.1038/sj.ejcn.1600528

[41] Rahkonen O, Lundberg O, Lahelma E, Huuhka M (1998) Body mass and social class: a comparison of Finland and Sweden in the 1990s. J Public Health Policy 19: 88–105. 9581432

[42] Welon Z, Szklarska A, Bielicki T, Malina RM (2002) Sex differences in the pattern of age-dependent increase in the BMI from 20–59 years. Am J Hum Biol 14: 693–698. 12400028 10.1002/ajhb.10079

[43] Lee HY, Choi CS, Birkenfeld AL, Alves TC, Jornayvaz FR, et al (2010) Targeted expression of catalase to mitochondria prevents age-associated reductions in mitochondrial function and insulin resistance. Cell Metab 12: 668–674. 21109199 10.1016/j.cmet.2010.11.004 PMC3013349

[44] Peterson CM, Johannsen DL, Ravussin E (2012) Skeletal muscle mitochondria and aging: a review. J Aging Res 2012: 194821. 22888430 10.1155/2012/194821 PMC3408651

[45] Wichmann HE, Gieger C, Illig T (2005) KORA-gen–resource for population genetics, controls and a broad spectrum of disease phenotypes. Gesundheitswesen 67 Suppl 1: S26–30. 16032514 10.1055/s-2005-858226

[46] Holle R, Happich M, Lowel H, Wichmann HE (2005) KORA–a research platform for population based health research. Gesundheitswesen 67 Suppl 1: S19–25. 16032513 10.1055/s-2005-858235

[47] Yang HC, Huang MC, Li LH, Lin CH, Yu AL, et al (2008) MPDA: microarray pooled DNA analyzer. BMC Bioinformatics 9: 196. 18412951 10.1186/1471-2105-9-196 PMC2387178

[48] Macgregor S, Visscher PM, Montgomery G (2006) Analysis of pooled DNA samples on high density arrays without prior knowledge of differential hybridization rates. Nucleic Acids Res 34: e55. 16627870 10.1093/nar/gkl136 PMC1440945

[49] Flaquer A, Heinzmann A, Rospleszcz S, Mailaparambil B, Dietrich H, et al (2014) Association study of mitochondrial genetic polymorphisms in asthmatic children. Mitochondrion 14: 49–53. 24270090 10.1016/j.mito.2013.11.002

[50] Smyth GK, R Core T (2010) p.adjust (part of the R package “stats”).

[51] R Core Team (2013) R: A Language and Environment for Statistical Computing. Vienna, Austria: R Foundation for Statistical Computing.

