# Supplementary figures and images for: Mitochondrial Genetic Variants Identified to Be Associated with BMI in Adults
Source: PLoS One. 2014 Aug 25;9(8):e105116. doi: 10.1371/journal.pone.0105116 (PMC4143221; doi:10.1371/journal.pone.0105116)

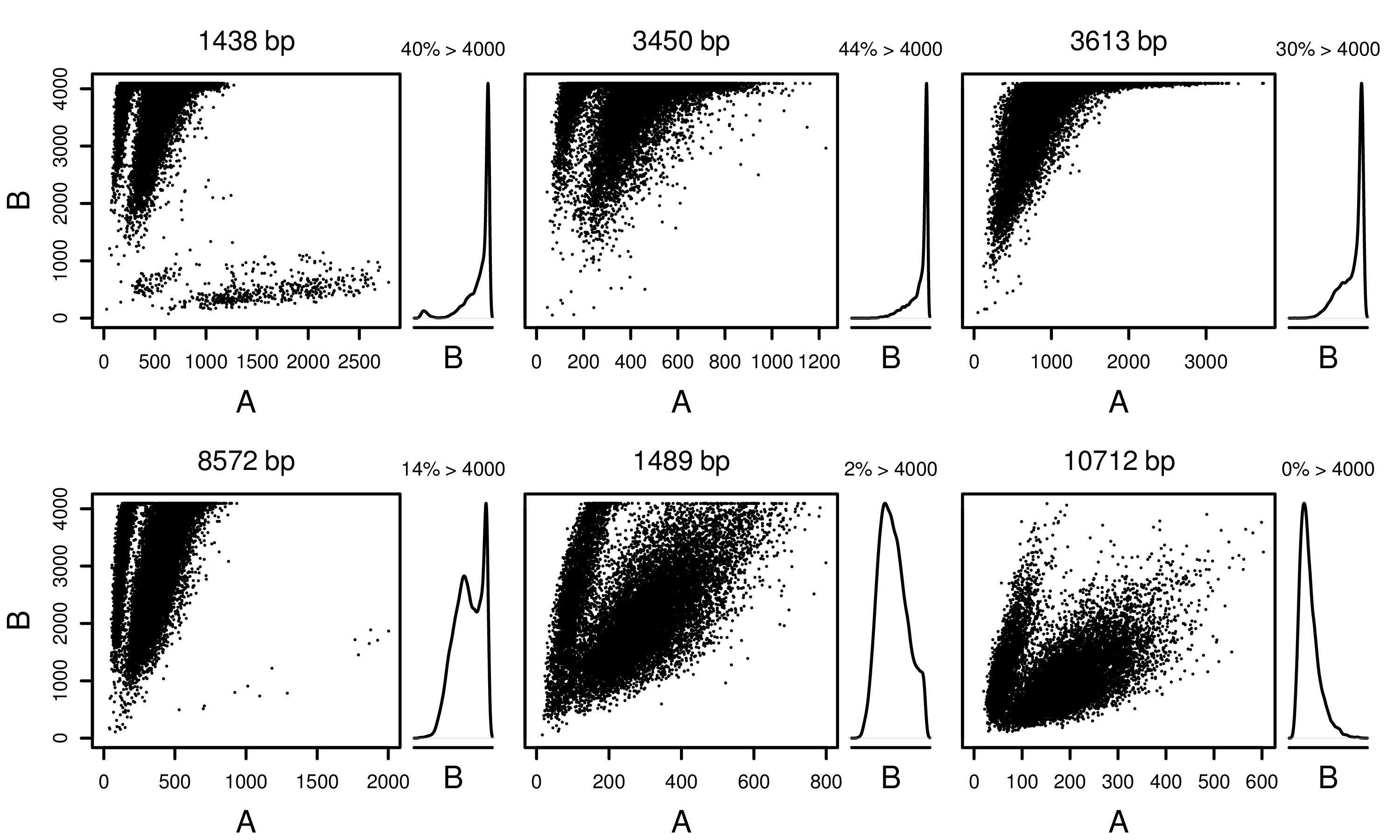

Supplement: Figure S1 — Examples of intensities affected by the cut-off. (TIFF) [file pone.0105116.s001.tiff]

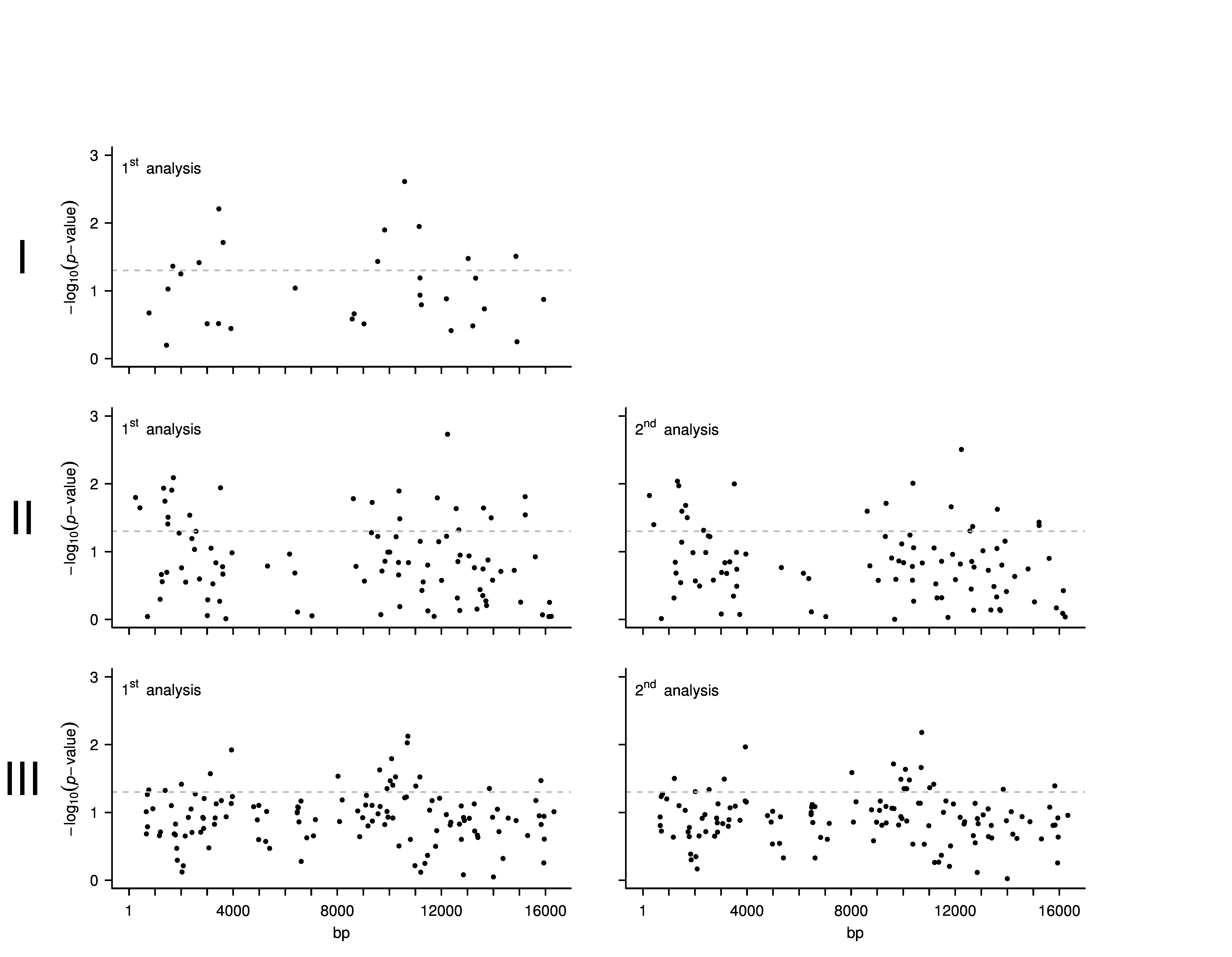

Supplement: Figure S2 — Resulting p-values from the 1st analysis (left side) and 2nd analysis (right side). (TIFF) [file pone.0105116.s002.tiff]
