# Supplementary material for: Mitochondrial Genetic Variants Identified to Be Associated with BMI in Adults
Source: PLoS One. 2014 Aug 25;9(8):e105116. doi: 10.1371/journal.pone.0105116 (PMC4143221; doi:10.1371/journal.pone.0105116)
Supplement: Table S1 — Estimates of the model parameters for mtSNPs with a Pnominal< = 0.01. Genomic position in base pairs (bp), alleles, rs_number, and point mutation are based on the NCBI dbSNP GRCh38 human genome assembly (rCRS, GeneBank ID J01415.2). Alleles are given in terms of major→minor allele. An estimated effect size (βSNP)<0 indicates that the risk allele is the minor allele. Nominal p-values and adjusted p-values are provided. μ: mean of log2(A/B). Covariate sex baseline: male. (DOC) [file pone.0105116.s003.doc]

|  | | | | **β estimates** | | | | **Std error** | | | | **P_nominal** | | | | **P_adjusted** |
| --- | --- | --- | --- | --- | --- | --- | --- | --- | --- | --- | --- | --- | --- | --- | --- | --- |
| **SNP_Name** | **bp** | **A→B** | **µ** | **β_0_** | **β_SNP_** | **β_SEX_** | **β_AGE_** | **β_0_** | **SNP** | **SEX** | **AGE** | **P_β_0_** | **P_β_SNP_** | **P_β_SEX_** | **P_β_AGE_** | **P_β_SNP_** |
| **CHIP: Affy.6.0** |  |  |  |  |  |  |  |  |  |  |  |  |  |  |  |  |
| rs28413696 | 4851 | C🡪T | -2.1 | 28.2 | -1.1 | -0.6 | 0.1 | 0.2 | 0.3 | 0.2 | 0.0 | <0.001 | **1.7E-04** | 4.9E-03 | 1.6E-09 | **2.9E-02** |
| rs9743 | 9698 | T🡪C | 3.0 | 28.2 | -0.2 | -0.7 | 0.1 | 0.2 | 0.1 | 0.2 | 0.0 | <0.001 | **2.2E-04** | 1.2E-03 | 7.2E-09 | **2.9E-02** |
| rs28416101 | 3336 | T🡪G | 5.7 | 28.2 | -0.2 | -0.6 | 0.1 | 0.2 | 0.1 | 0.2 | 0.0 | <0.001 | **2.6E-04** | 4.7E-03 | 4.6E-10 | **2.9E-02** |
| rs28358280 | 10550 | A🡪G | 1.7 | 28.2 | -0.3 | -0.7 | 0.1 | 0.2 | 0.1 | 0.2 | 0.0 | <0.001 | **2.8E-04** | 1.3E-03 | 1.4E-08 | **2.9E-02** |
| rs2854127 | 1780 | T🡪C | 2.7 | 28.2 | -1.1 | -0.7 | 0.1 | 0.2 | 0.3 | 0.2 | 0.0 | <0.001 | 1.0E-03 | 3.0E-03 | 2.1E-09 | 8.6E-02 |
| rs28429662 | 4926 | C🡪T | -4.2 | 28.2 | -0.5 | -0.7 | 0.1 | 0.2 | 0.2 | 0.2 | 0.0 | <0.001 | 1.6E-03 | 2.9E-03 | 9.2E-10 | 1.0E-01 |
| rs28358576 | 1811 | A🡪G | 2.1 | 28.2 | -0.2 | -0.7 | 0.1 | 0.2 | 0.1 | 0.2 | 0.0 | <0.001 | 1.9E-03 | 2.2E-03 | 1.6E-08 | 1.0E-01 |
| rs28502681 | 8572 | G🡪A | -0.2 | 28.2 | 0.6 | -0.7 | 0.1 | 0.2 | 0.2 | 0.2 | 0.0 | <0.001 | 2.2E-03 | 2.1E-03 | 1.3E-09 | 1.0E-01 |
| rs28441014 | 3972 | A🡪G | 2.1 | 28.2 | -1.5 | -0.7 | 0.1 | 0.2 | 0.5 | 0.2 | 0.0 | <0.001 | 2.5E-03 | 2.7E-03 | 1.0E-07 | 1.0E-01 |
| rs28550734 | 11840 | C🡪T | -2.5 | 28.2 | -0.5 | -0.7 | 0.1 | 0.2 | 0.2 | 0.2 | 0.0 | <0.001 | 2.5E-03 | 2.6E-03 | 3.8E-09 | 1.0E-01 |
| rs28408820 | 915 | C🡪A | -1.4 | 28.2 | -1.5 | -0.7 | 0.1 | 0.2 | 0.5 | 0.2 | 0.0 | <0.001 | 4.1E-03 | 2.7E-03 | 9.0E-09 | 1.5E-01 |
| rs3937033 | 16519 | C🡪T | -1.1 | 28.2 | 0.1 | -0.7 | 0.1 | 0.2 | 0.0 | 0.2 | 0.0 | <0.001 | 4.2E-03 | 2.3E-03 | 6.9E-09 | 1.5E-01 |
| rs28760274 | 2544 | C🡪T | -1.5 | 28.2 | -1.5 | -0.7 | 0.1 | 0.2 | 0.6 | 0.2 | 0.0 | <0.001 | 7.1E-03 | 2.2E-03 | 2.0E-08 | 2.0E-01 |
| rs28357678 | 14668 | C🡪T | -3.8 | 28.2 | 0.4 | -0.7 | 0.1 | 0.2 | 0.2 | 0.2 | 0.0 | <0.001 | 7.3E-03 | 3.2E-03 | 7.5E-09 | 2.0E-01 |
| rs3900944 | 14049 | G🡪A | -2.6 | 28.2 | -0.8 | -0.7 | 0.1 | 0.2 | 0.3 | 0.2 | 0.0 | <0.001 | 7.4E-03 | 3.7E-03 | 1.2E-09 | 2.0E-01 |
| rs28611051 | 3125 | A🡪G | 2.4 | 28.2 | -0.8 | -0.6 | 0.1 | 0.2 | 0.3 | 0.2 | 0.0 | <0.001 | 8.0E-03 | 5.0E-03 | 2.7E-08 | 2.1E-01 |
| rs28527344 | 1703 | C🡪A | -3.2 | 28.2 | -0.5 | -0.7 | 0.1 | 0.2 | 0.2 | 0.2 | 0.0 | <0.001 | 8.7E-03 | 3.9E-03 | 3.3E-09 | 2.1E-01 |
| rs35315169 | 16111 | C🡪T | -2.3 | 28.2 | -0.6 | -0.6 | 0.1 | 0.2 | 0.2 | 0.2 | 0.0 | <0.001 | 9.4E-03 | 4.6E-03 | 1.4E-08 | 2.2E-01 |
| **CHIP: Illum Exome** | |  |  |  |  |  |  |  |  |  |  |  |  |  |  |  |
| rs200784106 | 6663 | A🡪G | 6.6 | 27.7 | -0.5 | -0.7 | 0.1 | 0.1 | 0.1 | 0.2 | 0.0 | <0.001 | **6.2E-05** | 3.4E-05 | 6.3E-27 | **1.4E-02** |
| rs199771084 | 4021 | A🡪G | 3.5 | 27.7 | 1.0 | -0.7 | 0.1 | 0.1 | 0.3 | 0.2 | 0.0 | <0.001 | 1.4E-03 | 3.8E-05 | 1.9E-26 | 1.2E-01 |
| rs28357668 | 14148 | A🡪G | 5.3 | 27.7 | 0.2 | -0.7 | 0.1 | 0.1 | 0.1 | 0.2 | 0.0 | <0.001 | 1.6E-03 | 2.3E-05 | 4.9E-28 | 1.2E-01 |
| rs1116906 | 8460 | A🡪G | 3.6 | 27.7 | 0.6 | -0.7 | 0.1 | 0.1 | 0.2 | 0.2 | 0.0 | <0.001 | 4.2E-03 | 2.6E-05 | 1.3E-27 | 1.7E-01 |
| rs28357685 | 15110 | G🡪A | -1.7 | 27.7 | -0.7 | -0.7 | 0.1 | 0.1 | 0.3 | 0.2 | 0.0 | <0.001 | 4.5E-03 | 3.9E-05 | 1.1E-26 | 1.7E-01 |
| rs2853489 | 11172 | A🡪G | 3.6 | 27.7 | 0.8 | -0.7 | 0.1 | 0.1 | 0.3 | 0.2 | 0.0 | <0.001 | 4.6E-03 | 2.4E-05 | 3.7E-28 | 1.7E-01 |
| rs201212638 | 3398 | T🡪C | 8.9 | 27.7 | -0.1 | -0.7 | 0.1 | 0.1 | 0.0 | 0.2 | 0.0 | <0.001 | 6.1E-03 | 3.2E-05 | 2.5E-27 | 2.0E-01 |
| **CHIP: Affy. Axiom** | |  |  |  |  |  |  |  |  |  |  |  |  |  |  |  |
| rs28359170 | 12236 | G🡪A | 97.9 | 27.7 | -0.3 | -0.7 | 0.1 | 0.1 | 0.1 | 0.2 | 0.006 | <0.001 | 3.1E-03 | 1.2E-05 | 7.5E-48 | 2.6E-01 |
| rs3902408 | 10712 | C🡪T | 100.0 | 27.7 | -0.1 | -0.7 | 0.1 | 0.1 | 0.0 | 0.2 | 0.005 | <0.001 | 6.6E-03 | 7.8E-06 | 2.5E-48 | 2.6E-01 |
| rs28394599 | 1327 | G🡪A | 100.0 | 27.6 | -0.2 | -0.7 | 0.1 | 0.1 | 0.1 | 0.2 | 0.005 | <0.001 | 9.1E-03 | 9.3E-06 | 2.4E-48 | 2.6E-01 |
| rs28358277 | 10373 | G🡪A | 99.6 | 27.7 | -0.3 | -0.7 | 0.1 | 0.1 | 0.1 | 0.2 | 0.005 | <0.001 | 9.8E-03 | 1.0E-05 | 3.2E-48 | 2.6E-01 |
