# Supplementary material for: Mitochondrial Genetic Variants Identified to Be Associated with BMI in Adults
Source: PLoS One. 2014 Aug 25;9(8):e105116. doi: 10.1371/journal.pone.0105116 (PMC4143221; doi:10.1371/journal.pone.0105116)
Supplement: Text S1 — Quality control. (DOCX) [file pone.0105116.s004.docx]

**Text S1- Quality control**

Most of the time researchers analyze their genotype data after calling algorithms have already been applied, such that information on the raw intensity values is no longer available. As a consequence, in the vast majority of genetic projects data quality control is performed on the called data only, disregarding the original raw data. In this project, we analyzed mtSNPs and, in order to adequately take heteroplasmy into account, we were not directly interested in genotype calls but rather in the raw intensity values of mtSNPs.

First a visual check was performed plotting the intensity measures of each mtSNP separately for each chip. In this step only the Affymetrix Axiom chip displayed anomalies in some intensity values. There seems to be an upper bound for the intensity values at an intensity of 4095. As a result some mtSNPs had a substantial fraction of either their A or B intensities squeezed into the region just below that upper bound whereas without the bound those intensities apparently would have taken higher values. The effect corresponds to an artifact in the empirical density function of the intensities: a sharp peak in the upper tail. Some examples are illustrated in Figure S1.

**Special QC for the Affymetrix Axiom chip**

The manufacturer confirmed a technical reason for this behavior; the signal intensity plateau was due to scanner saturation. The intensities measured by the CCD camera which, is used for imaging on the Gene Titan Multichannel (GTMC), show plateaus at a signal intensity of about 4000. Because a list with SNPs likely to be affected could not be provided, and the possibility of repeating the genotyping was ruled out, we decided to apply a criterion to correct the cut-off effect. Afterwards, we tried to measure the effect of our correction when applied to those mtSNPs in our data with intensities affected by the cut-off problem.

In an attempt to smoothen the sharp peak in the density function we set intensities in the suspicious region between 4000 and 4095 to missing. Upon visual inspection we noticed that the peak was visibly smoothened except in SNPs that were strongly affected by the cut-off problem (more than 10% of either their A or B intensities affected with the cut-off). Therefore, we decided to remove from the analysis any mtSNPs that had more than 10% of the intensity values cut off to 4000. After this correction, chip-specific global background intensity was removed, intensities were quantile normalized, log_2_ (A_i_/B_i_) computed, and outliers removed as mentioned in the next section (cf. *Normalization of microarray data and outlier corrections*).

The Axiom chip contains a total number of 251 mtSNPs, of which 125 mtSNPs (49.8%) were affected by the cut-off problem. Applying our correction criteria, 31 mtSNPs (24.8% of the affected mtSNPs) needed to be completely removed from the analysis. The remaining 94 affected mtSNPs displayed less than 10% cut-off values. After setting those to missing, the artifact in the empirical density function disappeared, and so these mtSNPs could be retained for the analysis.

Since the Axiom chip provides multiple intensity measurements per SNP allele, we used mean A allele and mean B allele intensities and applied a linear regression model as described in the main text to assess mtSNP-phenotype association (cf. *Statistical methods).* In order to assess the effect of our cut-of correction criteria, the analysis was performed twice. In the first analysis all mtSNPs were included using the original intensity values, i.e., without correcting for the cut-off problem, while in the second analysis the intensity values were handled as mentioned above. Figure S2 represents the resulting p-values from the 1^st^ analysis (Figure A2, left side) and from the 2^nd^ analysis (Figure A2, right side). The results can be interpreted in three separated groups (I-III).

Group I contains those mtSNPs that were severely affected by the cut-off problem (>10% intensity values) and, based on our criteria, should be completely removed from the analysis. Therefore, these mtSNPs are considered only in the 1^st^ analysis (Figure A2-I). In this case, 10 out of 31 mtSNPs, i.e., 32.3%, resulted to be significant at a nominal α=0.05. For instance, mt3450 was associated with a p-value of 6.17x10^-04^. As can be observed in Figure S1, at 3450 bp, this mtSNP had intensities cut off for many individuals (44%). Hence, including mtSNPs in the analysis that are severely affected by the cut-off problem may apparently increase the number of false positive results.

Group II represents those mtSNPs for which setting intensity values > 4000 to missing succeeded in smoothing the upper tail (resulting in less than 10% missing values) and, based on our criteria, these mtSNPs were retained in the analysis. Differences between the observed p-values, P_1_ and P_2_, were detected between the 1^st^ and 2^nd^ analysis, respectively (Figure A2-II). These differences do not follow a pattern; some p-values increase while others decrease. For instance, at 10400 bp, P_1_ is equal to 0.03 and increases to P_2_ = 0.09, or at bp 1703, P_1_ is equal to 0.008 and increases to P_2_ = 0.03. When considering a 5% significance level, the first analysis may lead to a false positive finding for the mtSNPs at 10400 bp. When considering changes in significance, 7.3% of the loci happened to be significant in the 1^st^ analysis (P_1_≤0.05), but did not show significance in the 2^nd^ analysis (P_2_ >0.05). The opposite occurred in 5.7% of the loci (P_1_ >0.05 and P_2_ ≤0.05). Hence, leaving the cut-off values unchanged (1^st^ analysis) may inflate both the rate of false positive and false negative results.

Group III includes those mtSNPs that did not suffer from the cut-off problem. Even though the differences in p-values are not as large as in the second group some small variation is still observed (Figure 2A-III). Small differences between P_1_ and P_2_ are expected, despite the fact that these mtSNPs were not affected directly by the cut-off problem. They are indirectly affected by the background correction and normalization where all mtSNPs are considered together. Hence, the intensity values of the mtSNPs severely affected by the cut-off problem will influence unaffected mtSNPs. Note that the effect can have either direction, making the SNP more significant, less significant, or not affecting it at all. In this group only at one locus, 2036 bp, a change in the interpretation of significance was observed (P_1_=0.11 and P_2_= 0.002).

Altogether, based on our experience with analyses in which raw intensity data is handled, including SNPs affected by the cut-off problem may lead to an increase in the frequencies of both false positives and false negatives.

In this project we only used the raw intensity values from the mitochondrial genome. We did not investigate to what extent this cut-off problem affects nuclear SNPs and the corresponding quality of genotype calls. mtDNA is much more abundant in a (bio-) sample than nuclear DNA. Therefore, the observed intensities for mtSNPs are much higher than the intensities for nuclear SNPS, and so the cut-off problem may be more severe for mtSNPs than for nuclear SNPs. Anyway, a technical limitation exists in the scanning hardware for the Affymetrix Axiom chip which affects the raw intensity levels. This should be taken into account when performing statistical analysis.

**Normalization of microarray data and outlier correction**

Background correction is an important preprocessing step for microarray data that attempts to adjust the data for the ambient intensity surrounding each feature. In a second step, the intensities were quantile normalized applying the method proposed by Bolstad et al. [[1](#_ENREF_1)] and implemented in the R package limma [[2](#_ENREF_2)]. After quantile normalization, log_2_ intensity ratios, log_2_($\bar{A}/\bar{B}$), were computed for each individual. Since, due to the preceding quantile normalization, every individual has the same distribution of log_2_ intensity ratios, it is possible to make cross-individual comparisons and look for outlying observations on a per mtSNP basis. Our iterative outlier detection procedure consists of two steps: i) compute the interquartile range of log ratios disregarding missing values, ii) if the distance between the highest (smallest) and second highest (smallest) observation at a mtSNP is larger than the interquartile range, set the highest (smallest) observation to missing and go back to step i), otherwise proceed with the next mtSNP.

1. Bolstad BM, Irizarry RA, Astrand M, Speed TP (2003) A comparison of normalization methods for high density oligonucleotide array data based on variance and bias. Bioinformatics 19: 185-193.

2. Ritchie ME, Silver J, Oshlack A, Holmes M, Diyagama D, et al. (2007) A comparison of background correction methods for two-colour microarrays. Bioinformatics 23: 2700-2707.
